# Dysregulated lncRNAs are Involved in the Progress of Sepsis by Constructing Regulatory Networks in Whole Blood Cells

**DOI:** 10.3389/fphar.2021.678256

**Published:** 2021-08-17

**Authors:** Yanwei Cheng, Xue Cao, Jiange Zhang, Dong Chen, Juan Zhu, Lijun Xu, Lijie Qin

**Affiliations:** ^1^ Department of Emergency, Henan Provincial People’s Hospital, People’s Hospital of Zhengzhou University, People’s Hospital of Henan University, Zhengzhou, China; ^2^ Department of Rheumatology and Immunology, Henan Provincial People’s Hospital, People’s Hospital of Zhengzhou University, People’s Hospital of Henan University, Zhengzhou, China; ^3^ ABLife BioBigData Institute, Wuhan, China

**Keywords:** sepsis, transcriptome, lncRNA, mRNA, network

## Abstract

Sepsis is a highly heterogeneous syndrome that is caused by an unbalanced host response to an infection. Long noncoding RNAs (lncRNAs) have been reported to exert regulatory roles in a variety of biological processes, and became potential biomarkers and therapeutic targets for diverse diseases. However, current understanding on the roles of lncRNAs in sepsis is extremely limited. Herein, to decipher the underlying functions of lncRNAs, we reexplored the 83 transcriptome datasets from specimens with sepsis, no_sepsis by final diagnosis, and control. The results of differentially expressed genes (DEGs), differentially expressed lncRNA (DElncRNA) analysis, and co-expression analysis of lncRNA–mRNA pairs were obtained. We found that the expression pattern of lncRNAs was significantly activated in sepsis specimens, which was clearly distinguished in sepsis from no_sepsis and control specimens. By performing co-expression analysis, we found DElncRNAs were closely related to T-cell activation and immune response–related terms in sepsis by regulating mRNA expression in the *trans* manner. The lncRNA–mRNA network and the qRT-PCR test revealed that lncRNAs *LINC00861*, *RP11-284N8.3*, and *CTB-61M7.2* were significantly correlated with the pathogenesis of sepsis. In addition, weighted gene co-expression analysis (WGCNA) and *cis*-regulation analysis also revealed sepsis-specific lncRNAs were highly associated with important biological processes correlated with sepsis. In summary, the systematic dysregulation of lncRNAs is tightly involved in the remodeling of gene expression regulatory network in sepsis, and the lncRNA–mRNA expression network may be used to refine biomarker predictions for developing novel therapeutic approaches in sepsis.

## Introduction

Sepsis is defined as a life-threatening multi-organ dysfunction caused by a dysregulated host response to infection (Singer et al., 2016). Despite continuous progress in earlier recognition and treatment, unfortunately sepsis remains the leading cause of death in the critically ill patient population, with few therapeutic options due to early-stage uncontrolled inflammation together with late-stage protracted immunosuppression (Dombrovskiy et al., 2007; Melamed and Sorvillo, 2009; van der Poll et al., 2017). Annually, over 30 million people are affected by sepsis, and approximately 30% of those affected die (Fleischmann et al., 2016; Cheng et al., 2018). It should be noted that patients who survive sepsis often suffer from a poor long-term outcome with risk of cognitive and physical impairments (Iwashyna et al., 2010). In light of this fact, sepsis has been described as the quintessential medical disorder of the twenty-first century (Reinhart et al., 2017). In such a context, substantial research progress has been made in recent years to understand the basic mechanisms underlying sepsis pathogenesis and to identify potential biomarkers and targets for use as diagnostic, therapeutic, and prognostic tools. Of note, special attention has been paid to investigate the roles of long noncoding RNAs (lncRNAs) in sepsis.

LncRNAs are defined as a class of nonprotein-coding transcripts with a length of more than 200 nucleotides (Kung et al., 2013). Due to the properties of participating in chromatin rearrangement, histone modification, and modification of alternative splicing genes, as well as the regulation of gene expression, lncRNAs have emerged as important factors contributing to the development and progression of various diseases (Esteller, 2011; Schonrock et al., 2012; Beermann et al., 2016; Gu et al., 2018; Zhang et al., 2019a). As a benefit from the advance of next‐generation sequencing technology, a limited number of sepsis-associated lncRNAs had been identified (Cui et al., 2014; Du et al., 2017; Pellegrina et al., 2017; Zhang et al., 2019b; Bai et al., 2020). Geng et al. (Geng et al., 2019) demonstrated that lncRNA *MALAT1* expression was elevated in adults with sepsis and suggested lncRNA *MALAT1* could be developed as a potential biomarker for facilitating diagnosis and management in sepsis. Likewise, high lncRNA *NEAT1* expression showed close correlation with increased disease risk, enhanced severity, and higher 28-day mortality in sepsis (Yang et al., 2020). Bai et al. (Bai et al., 2020) also identified four pediatric sepsis-associated lncRNAs, including *RP11-1220 K2.2.1–7*, *ANXA3–2*, *TRAPPC5–1*, and *ZNF638–1*. These lncRNAs were significantly highly expressed in septic children and could be novel biomarkers for pediatric sepsis. As is well known, lncRNAs can interact with miRNAs or mRNAs to involve in the pathogenesis of diverse diseases. Wei et al. (Wei et al., 2020) provided experimental evidence that the upregulation of lncRNA *NEAT1* could interact with miR-144-3p to aggravate sepsis-induced myocardial cell injury through the NF-κB signaling pathway. Significantly, Cheng and his colleagues (Cheng et al., 2020) recently found five lncRNAs (*FENDRR*, *MALAT1*, *TUG1*, *CRNDE*, and *ANCR*) were correlated with sepsis-associated mRNA modules perhaps by acting as competing endogenous RNAs (ceRNAs). However, there have been only limited studies to investigate the lncRNA–mRNA expression network in sepsis, and the roles of sepsis-associated lncRNAs have not been fully elucidated, which may impede further study on the complex molecular mechanism of this deadly disease.

In the present study, our objectives are to 1) profile the differentially expressed (DE) lncRNAs and mRNAs expressed in sepsis based on a public RNA-seq data, 2) construct a novel co-expression network for DElncRNAs and DEmRNAs, 3) predict the target genes of *trans*- and *cis*-acting lncRNAs and their functions, and 4) identify some potential key pairs of lncRNAs and *trans*-targets in blood samples from septic patients for a future study of sepsis. These findings will contribute to a comprehensive understanding of the involvement of lncRNAs in sepsis and provide potential candidate lncRNAs for the diagnosis and treatment of sepsis.

## Materials and Methods

### Retrieval and Processing of Public Data

Public sequence data files of GSE63311 deposited by Pena et al. (Pena et al., 2014) were downloaded from the Sequence Read Archive (SRA) (https://www.ncbi.nlm.nih.gov/geo/). The GSE63311 dataset contains 83 RNA-seq samples in whole blood cells from 11 healthy controls and 72 suspected septic patients, who were retrospectively classified as “sepsis (n = 37)” and “no_sepsis (n = 35).” SRA run files were converted to fastq format with NCBI SRA Tool fastq-dump. The raw reads were trimmed of low-quality bases using a FASTX-Toolkit (v.0.0.13; http://hannonlab.cshl.edu/fastx_toolkit/). Finally, the clean reads were evaluated using FastQC (http://www.bioinformatics.babraham.ac.uk/projects/fastqc/).

### Reads Alignment and Differentially Expressed Gene Analysis

The high-quality clean reads were aligned to a complete human genome (GRCH38) by TopHat2 (Kim et al., 2013), allowing four mismatches. Uniquely mapped reads were ultimately used to calculate read number and reads per kilobase of exon per million fragments mapped (RPKM) for each gene. The expression levels of genes were evaluated using RPKM. The software edgeR (Robinson et al., 2010) was applied to screen the RNA-seq data for the differential expression of genes (DEGs). The results were analyzed based on the fold change (FC ≥ 2 or ≤ 0.5) and the false discovery rate (FDR ≤ 0.05) to determine whether a gene was differentially expressed.

### LncRNA Prediction and Direction Identification

**FIGURE 1:**
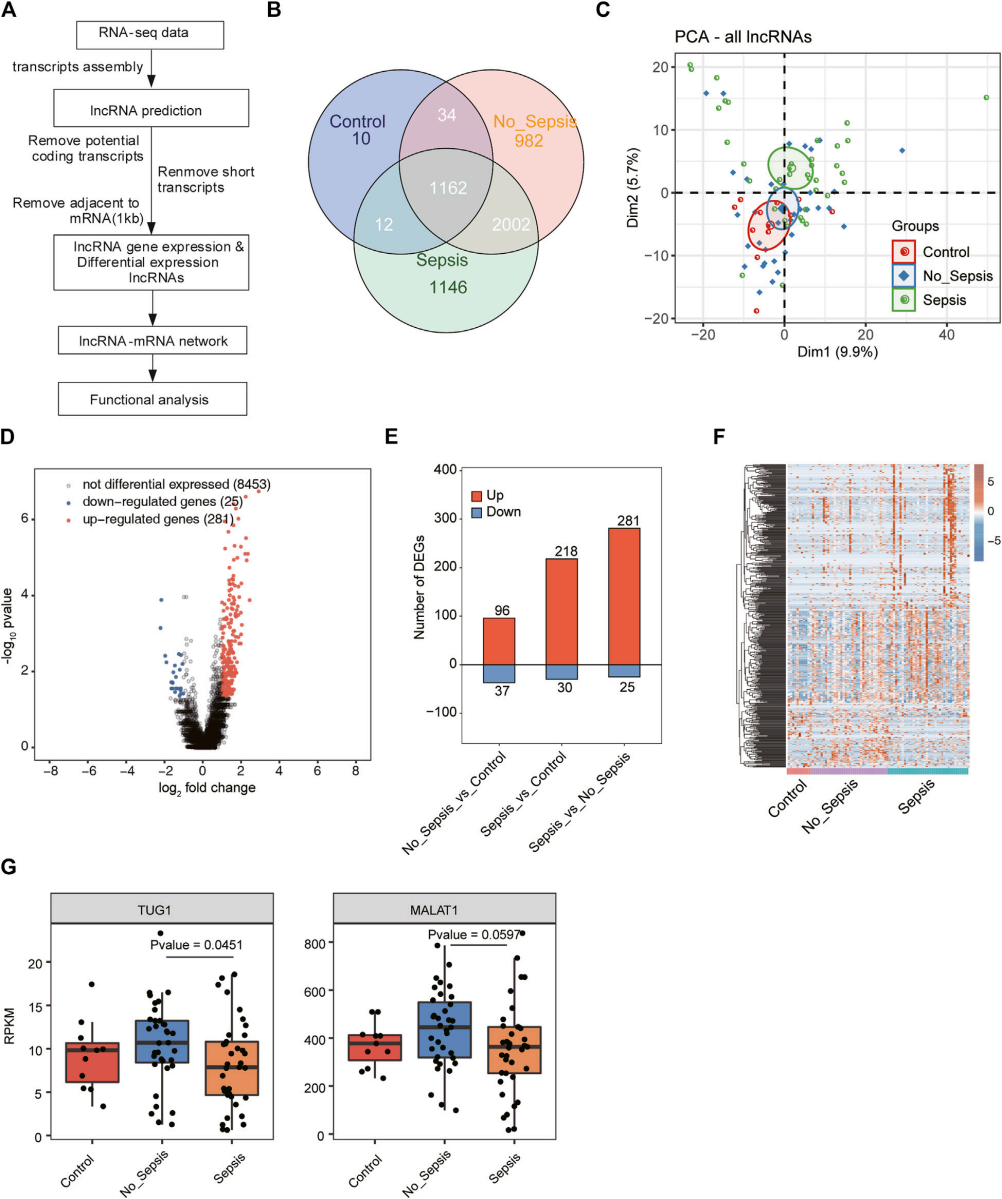
A comprehensive catalog and expression analysis of lncRNA genes in sepsis, no_sepsis, and control specimens. **(A)** Flowchart illustrated the experimental design and bioinformatic analysis pipeline for the identification and functional annotation of lncRNA genes expressed in sepsis, no_sepsis, and control specimens. **(B)** Venn diagram showed all the detected and overlapped lncRNAs in sepsis, no_sepsis, and control specimens. LncRNAs that have RPKM >= 0.2 in no less than two samples were considered as expressed in the group. **(C)** Principal component analysis (PCA) showed the distribution of all sepsis, no_sepsis, and control specimens based on the normalized expression level of all detected lncRNAs. The samples were grouped by Flowchart the disease state, and the ellipses for each group represent the confidence ellipse. **(D)** Volcano plot showed the distribution of DElncRNAs in the sepsis group compared with the no_sepsis group. Red and blue points indicate upregulated (FC > 2 and FDR < 0.05) and downregulated (FC < 0.5 and FDR < 0.05) genes, respectively. Black points indicated non-DElncRNAs. **(E)** Bar plot showed the number of upregulated (red) and downregulated (blue) DElncRNAs from the three comparison pairs. **(F)** Hierarchical clustering heatmap showed the normalized expression pattern of all DElncRNAs identified in **(E)**. **(G)** Box plot showed the expression levels of lncRNA *MATLA1* and *TUG1* in the RNA-seq dataset used in this study.

To systematically analyze the lncRNA expression pattern, we used a pipeline for lncRNA identification similar as previously reported (Liu et al., 2017), which was constructed based on the cufflink software (Trapnell et al., 2012). All steps of the pipeline have been shown in Figure 1A.

### WGCNA and Co-Expression Analysis

To fully understand the gene expression pattern and to cluster genes that have similar expression pattern with default parameters, we applied weighted gene co-expression network analysis (WGCNA) (Langfelder and Horvath, 2008). All expressed genes were used as input data. Eigengenes for each clustering module were used as the representative expression pattern of genes in each module. To explore the regulatory mode between lncRNAs and their host mRNAs, we calculated Pearson’s correlation coefficients (PCCs) between them and classified their relation into three classes: positive correlated, negative correlated, and non-correlated, based on the PCC value. *p*-value < 0.01 and absolute PCC > 0.6 between lncRNAs and mRNAs were picked out to draw networks by program Cytoscape (Shannon et al., 2003).

### Functional Enrichment Analysis

To sort out functional categories of DEGs, Gene Ontology (GO) terms and Kyoto Encyclopedia of Genes and Genomes (KEGG) pathways were identified using KOBAS 2.0 server (Xie et al., 2011). The hypergeometric test and the Benjamini–Hochberg false discovery rate (FDR) controlling procedure were used to define the enrichment of each term. Reactome (http://reactome.org) pathway profiling was also used for functional enrichment analysis of the sets of selected genes.

### Other Statistical Analysis

Principal component analysis (PCA) was performed by R package factoextra (https://cloud.r-project.org/package=factoextra) to show the clustering of samples with the first two components. After normalizing the reads by TPM (tags per million) of each gene in samples, in-house script (sogen) was used for visualization of next-generation sequence data and genomic annotations. The pheatmap package (https://cran.r-project.org/web/packages/pheatmap/index.html) in R was used to perform the clustering based on Euclidean distance. Student’s *t* test was used for comparisons between two groups.

### Validation of Gene Expression in RNA-Seq by qRT-PCR Analysis

In this study, to evaluate the validity of the lncRNA–mRNA expression changes in RNA-seq data, qRT-PCR was performed for the selected DElncRNAs and DEmRNAs. Whole blood samples were obtained from 19 healthy controls and 23 septic patients (Table 1 and Supplementary Table 1) in our hospital, which is a tertiary academic medical center with about 5,000 beds. This process was approved by the Ethics Committee of Henan Provincial People’s Hospital, as well as the agreement of all volunteers. We have strictly kept the standard biosecurity and institutional safety procedures in our country and area (Biosecurity Law of People’s Republic China). All the blood samples were processed immediately after collection for the isolation of peripheral blood mononuclear cells (PBMCs), which were stored at −80°C before RNA extraction.

**TABLE 1:** Clinical characteristics of the sepsis and control groups used in qRT-PCR validation.

Characteristic	Sepsis	Control	*p* value
Number	23	19	
Age, years, mean (SD)	70.30 (12.36)	27.58 (2.57)	< 0.0001^a^
Male, n (%)	15 (65)	12 (63)	> 0.9999^b^
Patients with pneumonia n (%)	18 (78)	—	
APACHE II score, median (IQR)	20 (17, 24)	—	
SOFA score, mean (SD)	7.04 (3.89)	—	

SD, standard deviation; IQR, interquartile range; APACHE, acute physiology and chronic health evaluation; SOFA, sequential organ failure assessment.

a *p* value of Student’s t test.

b *p* value of Fisher’s exact test.

First, total RNA from individuals subjected to sepsis (n = 23) and controls (n = 19) was extracted from PBMCs using the TRIzol reagent (Invitrogen) according to the manufacturer’s instructions. The RNA integrity of each sample was estimated using a 1.5% agarose gel electrophoresis and quantified by spectrometer. Then, 10 μg of the purified RNA was reverse-transcribed and taken for complementary DNA by PrimeScript RT Reagent Kit (Takara). Subsequently, qRT-PCR was conducted by using TB Green Fast qPCR Mix (Takara), specific primers (Table 2), and the following amplification conditions: denaturing at 95°C for 30 s, followed by 40 cycles of denaturing at 95°C for 10 s, and annealing and extension at 60°C for 30 s. Relative gene expression was determined by employing the 2^−ΔΔCT^ method and normalized against GAPDH. The Mann–Whitney *U* test was carried out to determine the expression difference between sepsis and control group. Statistical analyses were carried out using GraphPad Prism software (Mitteer et al., 2018). All *p*-values are two-sided. *p* < 0.05 was considered as statistically significant.

**TABLE 2:** Primer sequences used for qRT-PCR analysis.

LncRNA/mRNA	Forward primer	Reverse primer
CTB-61M7.2	5′-GTC​TTG​AAC​TCC​TGG​TCT​C-3′	5′-TGC​CTG​TAT​TCC​TTG​TAT​GA-3′
RP11-284N8.3	5′-GTC​CTC​CAC​TAA​TCA​CAG​AAT-3′	5′-TCA​CTT​GAT​GTC​AGA​ATG​CT-3′
LINC00861	5′-TGC​TCT​ACT​CCT​TGG​CTA​T-3′	5′-ACT​ACG​GTA​ACT​CCT​ATT​GC-3′
ACSL1	5′-GCA​GCA​AGT​AGC​AGA​CAT-3′	5′-GAA​GGA​AGC​GTT​CGT​GTT-3′
CD2	5′-AAG​AGC​CCA​CAG​AGT​AGC-3′	5′-GTG​GTG​GAG​GAG​GAT​GTT-3′
IL7R	5′-GAT​TCT​CTT​GCT​GCT​ACC​A-3′	5′-CGT​CTC​TTC​CGT​ATA​TCT​TCA-3′

## Results

### Genome-wide Profiling of the lncRNA Expression Associated with Sepsis

To investigate the expression change and molecular functions of lncRNAs in sepsis, we utilized the transcriptome sequencing data (RNA-seq) from 83 whole blood specimens (Pena et al., 2014), including 37 septic patients, 35 no_sepsis patients by final diagnosis, and 11 healthy controls. After obtaining the quality filtered reads, we aligned them to the human GRCH38 genome by TopHat2 (Kim et al., 2013). LncRNA prediction and functional analysis pipeline were conducted following a published method (Liu et al., 2017), which was illustrated in Figure 1A. After predicting novel lncRNAs and obtaining the expressed lncRNAs, we found the known lncRNAs occupied the most part of all lncRNAs (Supplementary Figures 1A,B). Most of the novel predicted lncRNAs were detected in all the three groups (Supplementary Figure 1A). Finally, we detected 1,218, 4,180, and 4,322 lncRNAs from control, no_sepsis, and sepsis groups, respectively. By comparing the exon number of lncRNAs and mRNAs, we found more than 90% of novel lncRNAs only had one exon, which was distinct from known lncRNAs and mRNAs (Supplementary Figure 1B). Length distribution analysis of lncRNAs and mRNAs also revealed the feature of lncRNAs that were shorter than mRNAs (Supplementary Figure 1C). We found 1,162 lncRNAs were detected in all the three groups, while only 10 specific lncRNAs were detected in the control group. Although a total of 3,164 lncRNAs were shared by the two sick groups, 982 and 1,146 lncRNAs were specifically detected in no_sepsis and sepsis groups, respectively (Figure 1B), indicating that lncRNAs were globally activated in sepsis and could distinguish sepsis from no_sepsis at initial clinical presentation.

To further explore the expression features of lncRNAs from these three groups, we performed principal component analysis (PCA) for both lncRNAs and mRNAs. Based on the expressed lncRNAs, we found sepsis and control specimens could be separated by the second component, while no_sepsis samples were in the middle (Figure 1C). Analysis based on the expressed mRNAs also showed similar results with lncRNAs (Supplementary Figure 1D), suggesting that the transcriptome profile was significantly changed between control/no_sepsis and sepsis specimens. We then performed differentially expressed lncRNA (DElncRNA) analysis for these three groups by edgeR (Robinson et al., 2010) with |log2 fold change| > 1 and a false discovery rate (FDR) < 0.05 as the criteria. By presenting the DElncRNA results between sepsis and no_sepsis, we found the upregulated DElncRNAs in sepsis were dominant (Figure 1D). DElncRNA statistical results also showed lncRNAs were globally upregulated in sepsis specimens, as well as no_sepsis specimens when compared with control (Figure 1E). Heatmap presentation of all the DElncRNAs also revealed the higher expression level of lncRNAs in sepsis and no_sepsis specimens (Figure 1F). In one previous study, five lncRNAs, including *FENDRR*, *MALAT1*, *TUG1*, *CRNDE*, and *ANCR*, were associated with sepsis (Cheng et al., 2020). We then analyzed their expression level in this dataset. Only *MALAT1* and *TUG1* were detected (RPKM > 0.2 in at least two samples of one group) in the RNA-seq data, and their expression levels were significantly different between no_sepsis and sepsis samples (Figure 1G). These results together indicated that lncRNAs were globally activated and showed distinct expression pattern in patients with sepsis.

### Co-Expression Analysis Between lncRNAs and mRNAs Revealed the Regulation Functions in a Trans Manner

**FIGURE 2:**
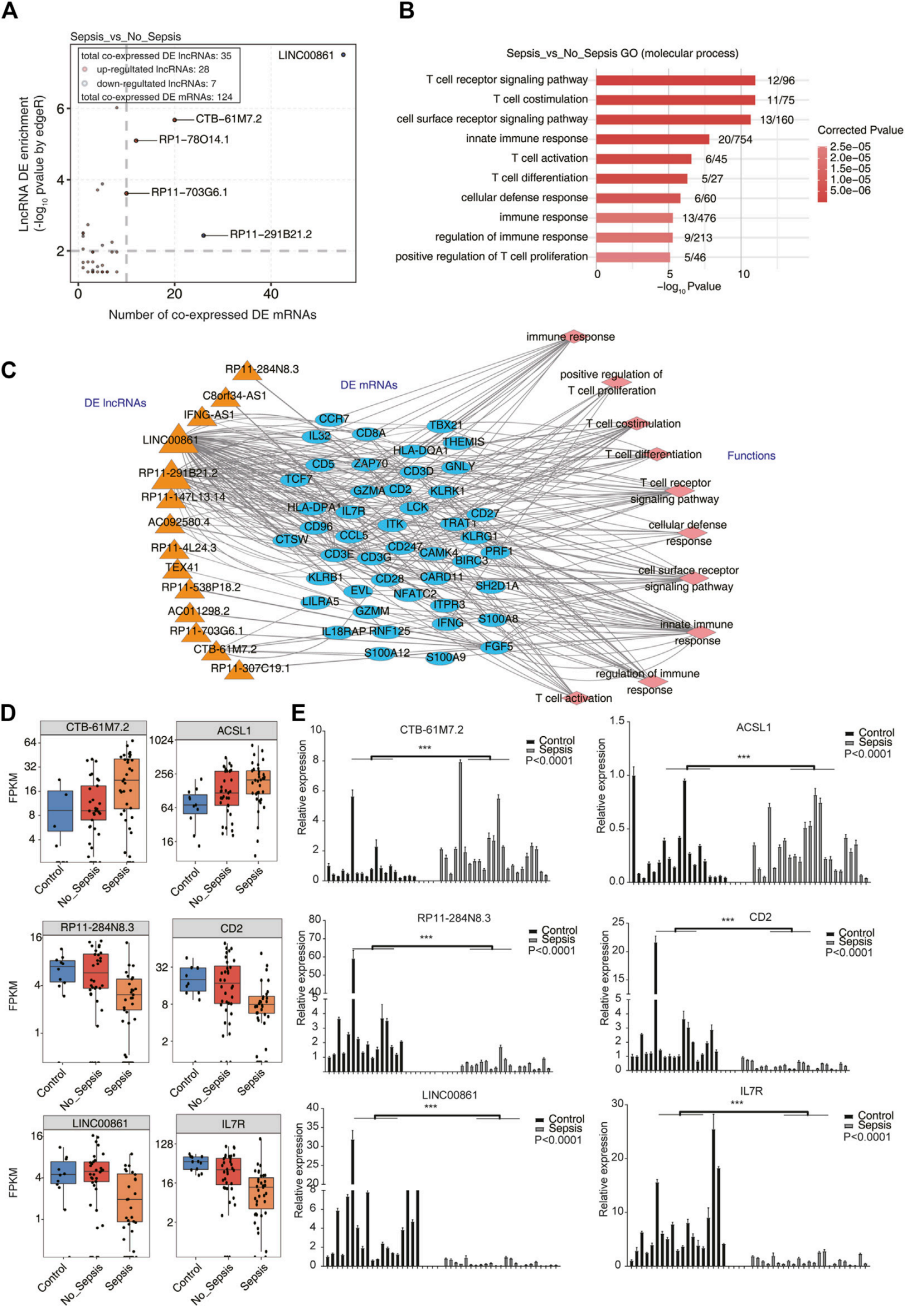
Co-expression network illustration between DElncRNAs and DEmRNAs. **(A)** Scatterplot showed the relationship between enrichment scores and co-expressed DEmRNA numbers of DElncRNAs in sepsis compared with no_sepsis samples. Red points denote upregulated lncRNAs involved in co-expression pairs, and blue points denote downregulated lncRNAs. Cutoffs of *p*-value < 0.01 and Pearson’s coefficient > 0.6 were applied to identify the co-expression pairs. **(B)** Bar plot showed the top 10 most enriched GO terms (biological process, BP) of the DEmRNAs co-expressed with DElncRNAs. (C) Co-expression network showed the interaction between DElncRNAs and DEmRNAs that were involved in the top 10 GO BP terms showed in **(B)**. DElncRNAs were on the left panel, their co-expressed DEmRNAs were in the center panel, and the DEmRNA-enriched GO BP terms were on the right panel. **(D)** Box plot showed the expression pattern of three lncRNA–mRNA pairs (*CTB-61M7.2-ACSL1*, *RP11-284N8.3-CD2*, and *LINC00861-IL7R*) from RNA-seq analysis results (GSE63311). **(E)** Bar plot showed the validation results of the three lncRNA–mRNA pairs in **(D)** by qRT-PCR experiment. ***p < 0.001, two-tail unpaired *t*-test.

To further explore the functions of DElncRNAs in sepsis, we performed co-expression analysis between lncRNAs and mRNAs based on the concept that genes with a correlated expression pattern may have similar functions or be regulated with each other in *a trans* manner. Pearson’s correlation coefficients (PCCs) were calculated between DElncRNAs and DEmRNAs for each pair. *p*-value < 0.01 and absolute PCC > 0.6 were set as the criteria to predict lncRNA–mRNA pairs. Functional analysis of co-expressed DEmRNAs was performed by Gene Ontology (GO) and Kyoto Encyclopedia of Genes and Genomes (KEGG) enrichment analyses. As a background, we first analyzed the DEmRNA–DEmRNA pairs and explored their functions. The results revealed immune response– and T-cell stimulation–related terms were enriched, which could represent the function of DEmRNAs (Supplementary Figure 2A). We finally obtained 241 lncRNA–mRNA pairs in sepsis *vs.* no_sepsis groups, including 35 DElncRNAs and 124 DEmRNAs. By plotting the co-expressed DEmRNA number and enrichment of DElncRNAs, we found several DElncRNAs were highly enriched (>2) and abundantly co-expressed with DEmRNAs (>10 DEmRNAs), including *LINC00861*, *CTB-61M7.2*, *RP1-78O14.1*, *RP11-703G6.1*, and *RP11-291B21.2* (Figure 2A). We found that co-expressed DEmRNAs were particularly enriched in T-cell activation– and immune response–related GO biological process (BP) terms (Figure 2B). KEGG enrichment analysis also revealed similar functional pathways (Supplementary Figure 2B). To find out the details of the lncRNAs co-expressed with mRNAs in Figure 2B, we generated a regulatory network between DElncRNAs and DEmRNAs using Cytoscape software (Shannon et al., 2003). We found 13 DElncRNAs and 46 DEmRNAs participated in the T-cell activation and immune response network (Figure 2C). Specially, lncRNA *LINC00861* was co-expressed with the most DEmRNAs in the T-cell activation and immune response network (Figure 2C). Using the same methods, we explored the DElncRNA–DEmRNA pairs and their functions in sepsis *vs.* control groups (Supplementary Figure 2C). Functional analysis of the co-expressed DEmRNAs in sepsis *vs.* control also revealed the T-cell activation– and immune response–related terms (Supplementary Figure 2D). Several lncRNAs, including *CTB-61M7.2*, *LINC00861*, and *RP11-284N8.3*, were detected in sepsis *vs.* no_sepsis and sepsis *vs.* control groups. We then plotted the expression level of the lncRNA–mRNA pair of *CTB-61M7.2-ACSL1* in these three groups and found they were significantly upregulated in sepsis specimens compared with control and no_sepsis (Figure 2D), whereas the two *RP11-284N8.3-CD2* and *LINC00861*–*IL7R* pairs were significantly downregulated in sepsis (Figure 2D). Taken together, these results suggested DElncRNAs in sepsis have potential roles in regulating the expression of genes involved in T-cell activation and immune response functions.

### Validation of lncRNA-Regulated Gene Expression Network by qRT-PCR

To validate the reliable expression changes of the sepsis-associated lncRNAs participated in *trans*-regulation detected in GSE63311 RNA-seq data, the three DElncRNAs (*CTB-61M7.2*, *RP11-284N8.3*, and *LINC00861*) and three highly co-expressed DEmRNAs (*ACSL1*, *CD2*, and *IL7R*) in the co-expression network (Figure 2C) were further selected for qRT-PCR validation using the blood samples collected from 19 normal controls and 23 patients with sepsis. The independent validation samples were chosen with matched gender information between control and sepsis individuals (Table 1 and Supplementary Table 1), while the age difference between control and sepsis samples was significant because of the unobtainable reason of old and healthy individuals in a short time. As presented in Figure 2E, the expression levels of the *RP11-284N8.3-CD2* and *LINC00861-IL7R* in sepsis group were significantly lower than those of control group, whereas the *CTB-61M7.2-ACSL1* was significantly upregulated in sepsis *vs.* control groups (*p* < 0.001 respectively). The qRT-PCR results were consistent to those acquired from RNA-seq in GSE63311 (Figure 2D), suggesting that expression levels of the three lncRNAs (*CTB-61M7.2*, *LINC00861*, and *RP11-284N8.3*) were changed in septic patients, and could be potential novel biomarkers and targets for facilitating diagnosis and management in sepsis.

Details of these results are provided in Supplementary Table 2.

### WGCNA Analysis of lncRNA–mRNA Co-expression Modules

**FIGURE 3:**
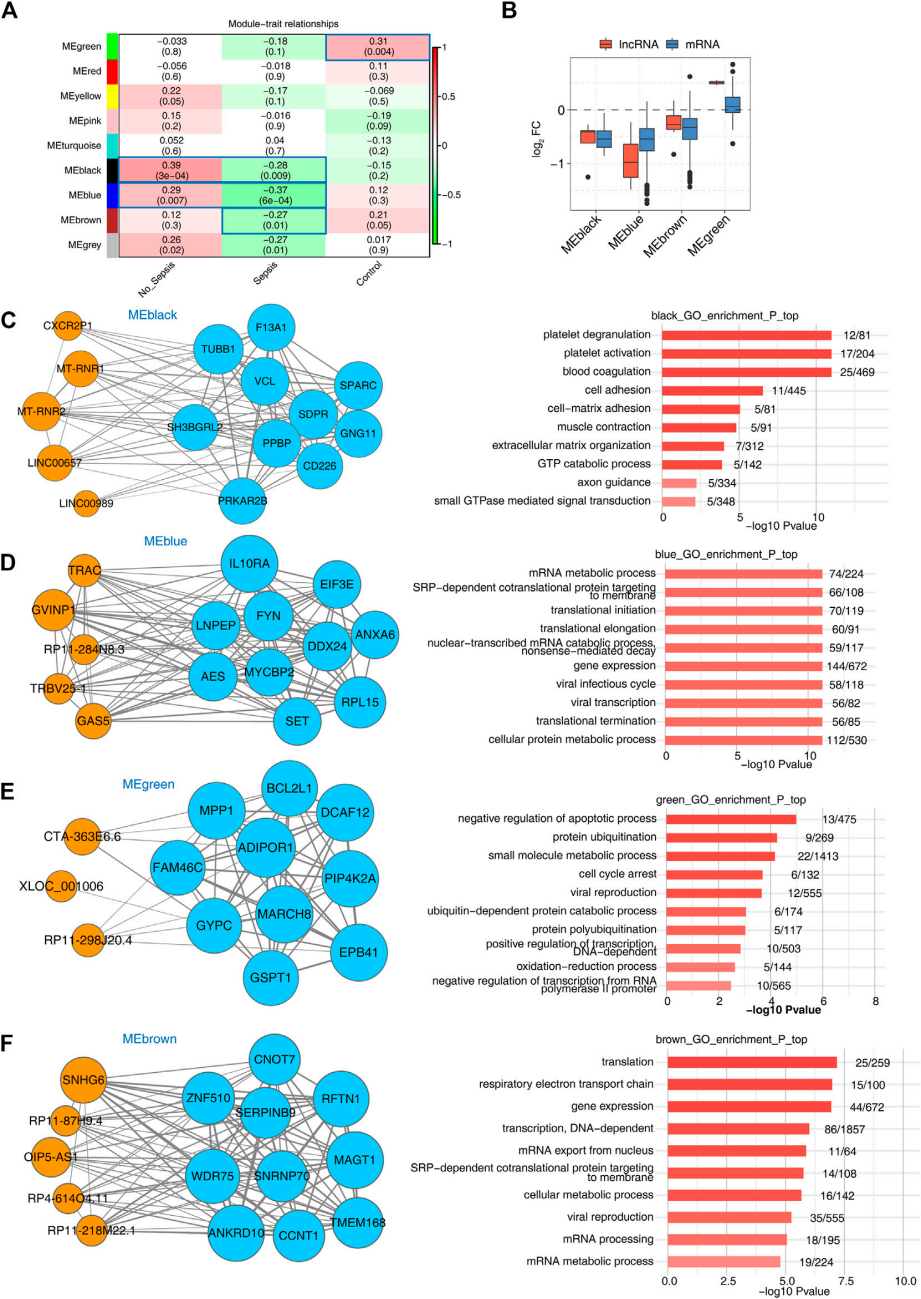
WGCNA analysis presentation of all expressed lncRNAs and mRNAs. **(A)** Heatmap plot showed the module-trait associations by a linear mixed effect (LME) model. Sample factors on the x axis were used as covariates. All Pearson’s correlation coefficient (PCC) values and statistical *p*-values (in the brackets) were displayed. Module-trait associations with *p*-values < 0.5 were framed with blue box. **(B)** Box plot showed the expression fold changes (sepsis *vs.* control group) of mRNAs and lncRNAs from the four disease-associated modules. **(C–F)** LncRNA–mRNA interaction network and enriched functional pathways showed the top hub lncRNA and mRNA interaction along with the enriched GO BP terms of the four modules, MEblack **(C)**, MEblue **(D)**, MEgreen **(E)**, and MEbrown **(F)**. Orange circles indicate lncRNAs and light blue circles indicate mRNAs.

Besides co-expression analysis, weighted gene co-expression network analysis (WGCNA) is another powerful method to decipher the underlying regulatory network between lncRNAs and mRNAs (Langfelder and Horvath, 2008). We combined the expression file of DElncRNAs and DEmRNAs together to perform WGCNA to obtain nine consistent expression modules, which contained 65 to 5,175 genes. Hierarchical clustering dendrogram showed the smaller distance within modules (Supplementary Figure 3A). Then module-trait association analysis was performed to explore the correlation between modules and specimen groups (trait). We found four modules, including black, blue, brown, and green, were significantly associated with the three groups. Green and brown modules were positively associated with the control group, while black and blue modules were positively associated with the no_sepsis group. All the four modules were negatively associated with sepsis group (Figure 3A). Eigengene bar plot presentation of these four modules showed higher expression level in no_sepsis group for black, blue, and brown modules, and higher expression level in control group for green module (Supplementary Figure 3B–E). We then calculated the fold change of lncRNAs and mRNAs from these four modules in sepsis *vs.* control groups. For black, blue, and brown modules, the log2 FC values of lncRNAs and mRNAs were both under zero. For green module, the log2 FC values of lncRNAs and most of the mRNAs were above zero (Figure 3B), demonstrating the distinct expression pattern among modules. We then integrated the co-expression results and functional analysis to identify the potential functions of lncRNAs in sepsis-associated modules. The regulatory network in black module revealed that five hub lncRNAs were co-expressed with ten mRNAs. Platelet degranulation and blood coagulation related functions were significantly enriched in black module (Figure 3C). For the blue module, mRNA metabolic process and translation-associated functions were enriched. Co-expression network including 5 hub lncRNAs and 10 mRNAs was shown in Figure 3D. The green module that was presented by three hub lncRNA and ten mRNA networks was enriched in the apoptotic process, protein ubiquitination, and cell cycle arrest–related functions (Figure 3E). The brown module that was presented by five hub lncRNAs, and ten mRNAs network was enriched in translation, respiratory electron transport chain, and gene expression related functions (Figure 3F). From the WGCNA results, we detected sepsis-associated modules that contained lncRNAs and mRNAs with specific functions, which perhaps influences the development of sepsis.

### Cis-Acting Analysis of lncRNA Regulation in Sepsis

**FIGURE 4:**
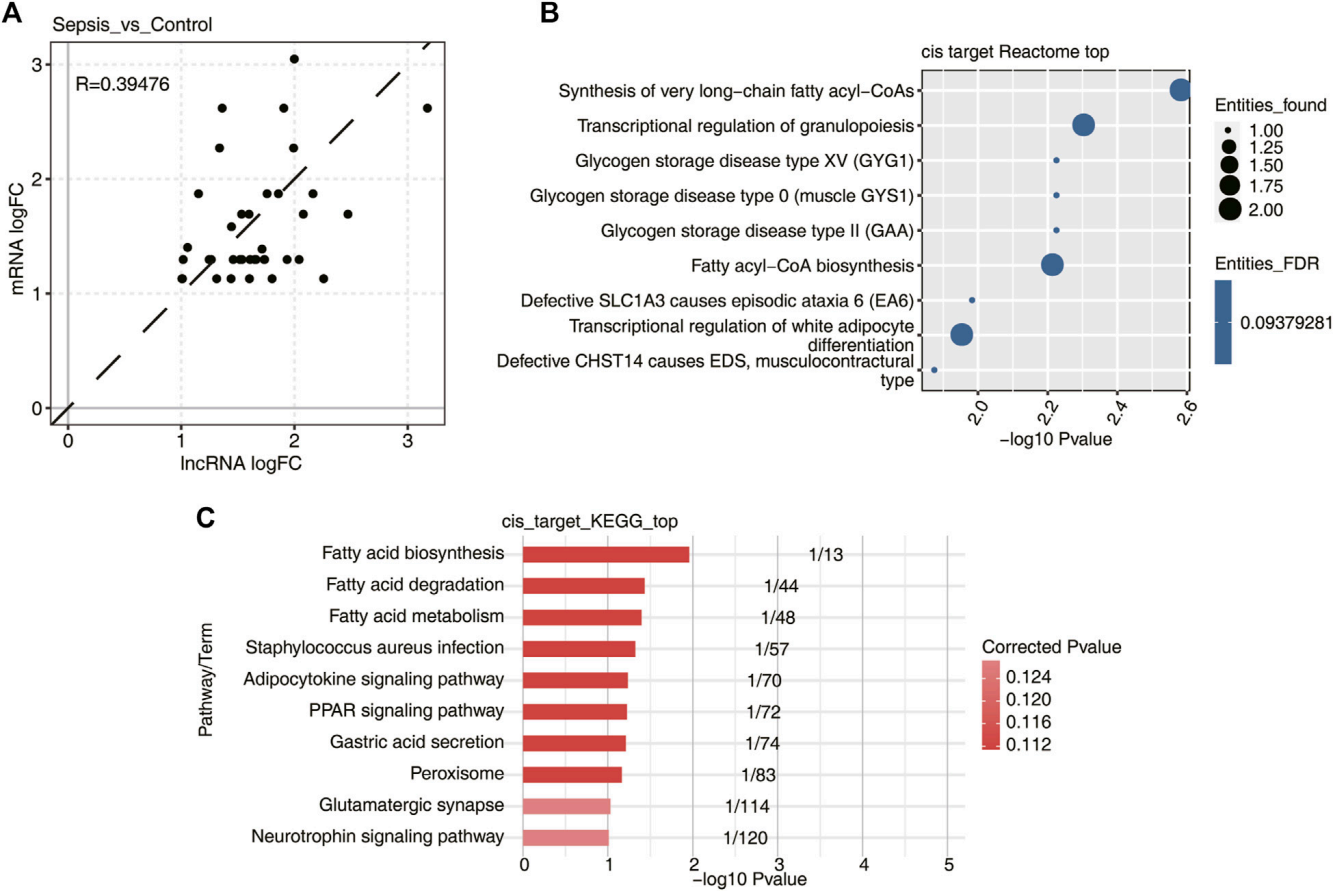
DElncRNAs and their potential mRNA targets by *cis*-regulatory prediction. **(A)** Scatterplot showed the log2 fold change (log2 FC) correlation between DElncRNAs and their *cis*-regulatory genes in sepsis *vs.* control samples. Dotted black line indicated the 45° line. **(B)** Bubble plot showed the top nine most enriched Reactome pathways of DElncRNA *cis*-regulated genes. **(C)** Bubble plot showed the top 10 most enriched KEGG pathways of DElncRNA *cis*-regulated genes.

Previous studies have confirmed that many lncRNAs regulate the expression of neighboring protein-coding genes through *cis*-acting mechanisms. These *cis*-acting lncRNAs recruit various transcription factors or chromatin remodeling complexes to change the transcription status of nearby genes. Accordingly, the expression levels between those lncRNAs and their neighboring genes are highly correlated. To test whether the DElncRNAs identified in sepsis behave in a similar way, a total of 233 DElncRNAs, together with 238 mRNAs in the upstream or downstream 10 kb range of DElncRNAs, were selected and subjected to co-expression analysis. Finally, 21 lncRNA–mRNA pairs with *p*-value < 0.01 and absolute PCC > 0.6, probably associated with *cis*-acting regulation, were identified in sepsis vs. control groups. Surprisingly, the expression level of these DElncRNAs with FC > 2 were positively associated with those of DEmRNAs (Figure 4A), suggesting lncRNAs might function as a positive regulator of mRNAs with significant co-expression in the development of sepsis. Subsequently, functional enrichment analysis was performed for the DEmRNAs highly co-expressed with DElncRNAs by sepsis compared with control samples, and they were significantly enriched in fatty acid metabolism and granulopoiesis related GO reactome terms (Figure 4B). We also analyzed these DEmRNAs *cis*-regulated by DElncRNAs using the KEGG pathway (Figure 4C). The most highly represented pathways included fatty acid biosynthesis, degradation, and metabolism. Together, these findings strongly indicated that the *cis*-acting lncRNAs might play major roles in regulation of their adjacent genes related to fatty acid metabolism and granulopoiesis in response to sepsis. Future studies to elucidate the connection between the lncRNA-neighboring gene pairs are essential to understand the working mechanism of these lncRNAs.

## Discussion

Sepsis, severe life-threatening infection with multi-organ dysfunction, initiates a complex immunopathogenic process that involves both innate and adaptive immune cells. In a real sense, sepsis can be considered a race to the death between the pathogens and the host immune system. With the development of sequencing technology, previous studies have demonstrated that the activation of immune cells by pathogens leads to rapid and dynamic changes in gene expression aimed at combating the infection. Notably, lncRNAs are linked and appear as a variational trend during the pathogenesis of sepsis. However, only a few studies have investigated the relationship between lncRNAs and sepsis, and the roles of lncRNAs in sepsis has not been fully elucidated. Future studies are required to investigate how lncRNAs influence sepsis and regulate the exact molecular signaling pathways, as well as to discover candidate lncRNAs for diagnosis and treatment.

In the present study, a total of 9,720 lncRNAs were detected in the RNA-seq data from 83 whole blood specimens, including 37 septic patients, 35 no_sepsis patients, and 11 healthy controls. Of these, 1,146 lncRNAs were specifically detected in sepsis groups, suggesting that lncRNAs were globally activated in sepsis samples. Previous studies have suggested that lncRNAs may be potential biomarkers for prognosis of sepsis, such as lncRNAs *MALAT1*, *NEAT1*, *RP11-1220 K2.2.1–7*, *ANXA3–2*, *TRAPPC5–1*, and *ZNF638–1* (Chen et al., 2018a; Geng et al., 2019; Bai et al., 2020), which have been introduced in introduction. Herein, the PCA result showed sepsis and control specimens could be separated by the second component, providing the solid evidence that lncRNAs could be diagnostic biomarkers to distinguish sepsis from no_sepsis and control specimens.

Emerging evidences have demonstrated that lncRNAs can act in a *trans* manner to affect the expression of genes located far away (Huarte et al., 2010; Fukuda et al., 2019). Therefore, in this study, a co-expression network analysis was performed to predict the functions of lncRNAs based on the enriched functions of their co-expressed genes. Totally, 44 DElncRNAs, together with 162 co-expressed DEmRNAs, were identified in sepsis, no_sepsis, and control groups. Enrichment analysis revealed that these *trans*-acting lncRNAs were mainly involved in T-cell activation and immune response–related terms, which were well known to be associated with occurrence and development of sepsis (van der Poll et al., 2017). Importantly, we identify three potential key pairs of lncRNAs and target mRNAs, including *LINC00861-IL7R*, *RP11-284N8.3-CD2*, and *CTB-61M7.2-ACSL1*, which were detected ubiquitously in three groups. IL-7R is the receptor of IL7*,* and the involvement of IL7 in sepsis is well recognized (Guimond et al., 2009; Mackall et al., 2011; Hotchkiss et al., 2013; Cui et al., 2014; Cruz-Zárate et al., 2018). IL7R can mediate the effects of IL7, which is indispensable for the growth, differentiation, and effector functions of T cells (Kalman et al., 2004; Cruz-Zárate et al., 2018). McGlynn et al. (Unsinger et al., 2010) showed that the increased IL7R expression in T cells likely serves to enhance IL7’s effect and improves survival in sepsis, suggesting that IL7R is a crucial T-cell developmental pathway. Prior studies had showed that lncRNA *LINC00861* was related to cancers (Zhang et al., 2017; Lv et al., 2018; Zheng et al., 2020; Liu et al., 2021), chronic obstructive pulmonary disease (Qian et al., 2018), atrial fibrillation (Ruan et al., 2020), and Parkinson’s disease (Lei et al., 2020; Liu et al., 2021). Our works strongly suggested that the deregulation of lncRNA *LINC00861* correlated with *IL7R* might promote sepsis development through mediating functions of T cells. Similar to the pair of *LINC00861*–*IL7R*, we found the expression levels of *RP11-284N8.3-CD2* in the sepsis group were significantly lower than those of control and no_sepsis groups. CD2 is a transmembrane glycoprotein of the immunoglobulin superfamily expressed on virtually all T and natural killer cells (McNerney and Kumar, 2006; Binder et al., 2020). It is known to participate in a co-stimulatory pathway of T-cell activation (Suthanthiran, 1989; Erben et al., 2015). CD2 plays an important role in NK cell conjugation to target cells as well (McNerney and Kumar, 2006; Paul and Lal, 2017). This result suggested that lncRNA *RP11-284N8.3* could play a role in sepsis. Up to now, there is no report on this novel lncRNA in any disease, and our work filled the gap in knowledge of lncRNA *RP11-284N8.3*. Instead, we observed that the lncRNA–mRNA pair of *CTB-61M7.2-ACSL1* was significantly upregulated in sepsis specimens compared with control and no_sepsis. ACSL1, a main member of long-chain acyl-CoA synthetases (ACSL) family, is an enzyme that converts fatty acids to acyl-CoA-derivatives for use in both lipid catabolism and lipid synthesis (Mashek et al., 2007). Besides exerting a role in the pathogenesis to fatty liver disease, obesity, atherosclerosis, diabetes, neurological disorders, and specific types of cancer (Yan et al., 2015), ACSL1 palys a likely role in driving inflammasome-mediated release of pro-inflammatory factors by neutrophils during sepsis, based on both profiling of the literature (Al-Rashed et al., 2019; Kalugotla et al., 2019; Roelands et al., 2019) and transcript co-expression analysis (Cheng et al., 2016). Taken together, these results indicated that the *trans*-acting DElncRNAs of the co-expression network involved in the pathogenesis of sepsis mainly through participating in T cell activation and immune response. In addition, significantly different expressions between sepsis patients and controls were validated by qRT-PCR for the aforementioned three pairs of lncRNAs–mRNAs, validating the results acquired from RNA-seq data. Although we validated their expression with not so large samples (23 sepsis *vs.* 19 controls), their expression difference was significant and consistent compared with the published dataset. Another shortage of validation is the age difference between sepsis and control samples as sepsis is an age-associated disease (Starr and Saito, 2014), which could be remedied by providing age matched samples in future study. These results suggest that lncRNAs *LINC00861, RP11-284N8.3, and CTB-61M7.2* could be potential candidates as biomarkers for sepsis and need to be further evaluated and investigated using more clinical samples and advanced diagnostic methods to put forward their clinical values.

LncRNAs can also execute their functions in *cis*-acting to regulate the expression of their adjacent genes (Ding et al., 2019). Notably, Pellegrina et al. (Pellegrina et al., 2017) did not observe any significant association between the expression of lncRNAs and neighboring DEmRNAs in sepsis. Besides, Cui et al. (Cui et al., 2014) determined whether *lncIL7R* regulates lipopolysaccharide (LPS)-induced inflammatory response via *cis*-regulating the expression of IL7R, the coding gene that overlaps with *lncIL7R*. However, *lncIL7R* appeared to function independently of IL7R in regulation of inflammatory response to LPS. Here, we identified a total of 16 *cis*-acting lncRNA–mRNA pairs in sepsis vs. control groups. These lncRNAs might function as a positive regulator of mRNAs, which were mainly involved in fatty acid metabolism and granulopoiesis process (Paudel et al., 2019; Brook et al., 2020), closely related to sepsis (Chen et al., 2018b; Das, 2019; Pradelli et al., 2020). Determining novel *cis*-acting sepsis-associated lncRNAs is an interesting issue that is worth pursuing in the future.

## Conclusion

In summary, we identified a set of differentially expressed lncRNAs and mRNAs in patients with sepsis. The co-expression network of these lncRNAs and mRNAs was constructed to explore functions of DElncRNAs; the results of which suggested that lncRNAs in the co-expression network were involved in the pathogenesis of sepsis. Combining with bioinformatic analysis, literature reports, and sample verification, we have screened three potential key pairs of lncRNA and target mRNA, and hypothesized that these potential key lncRNAs may *trans*-act with their corresponding coding genes to regulate T-cell activation and immune response. However, further studies should be carried out to verify the association between these lncRNAs and target mRNAs, and whether these lncRNA–mRNA axes play an important role in the development of sepsis.

## Data Availability

Publicly available datasets were analyzed in this study. These data can be found here: https://www.ncbi.nlm.nih.gov/geo/, using accession number GSE63311.

## Fundings

The present work was supported by the 23456 Talent Project of Henan Provincial People’s Hospital to LQ, Research Startup fund of Henan Provincial People’s Hospital to YC and XC, and Joint project of medical teaching and research to JZhu (Wjlx2020044).
