# Supplementary figures and images for: Dysregulated lncRNAs are Involved in the Progress of Sepsis by Constructing Regulatory Networks in Whole Blood Cells
Source: Front Pharmacol. 2021 Aug 17;12:678256. doi: 10.3389/fphar.2021.678256 (PMC8416166; doi:10.3389/fphar.2021.678256)

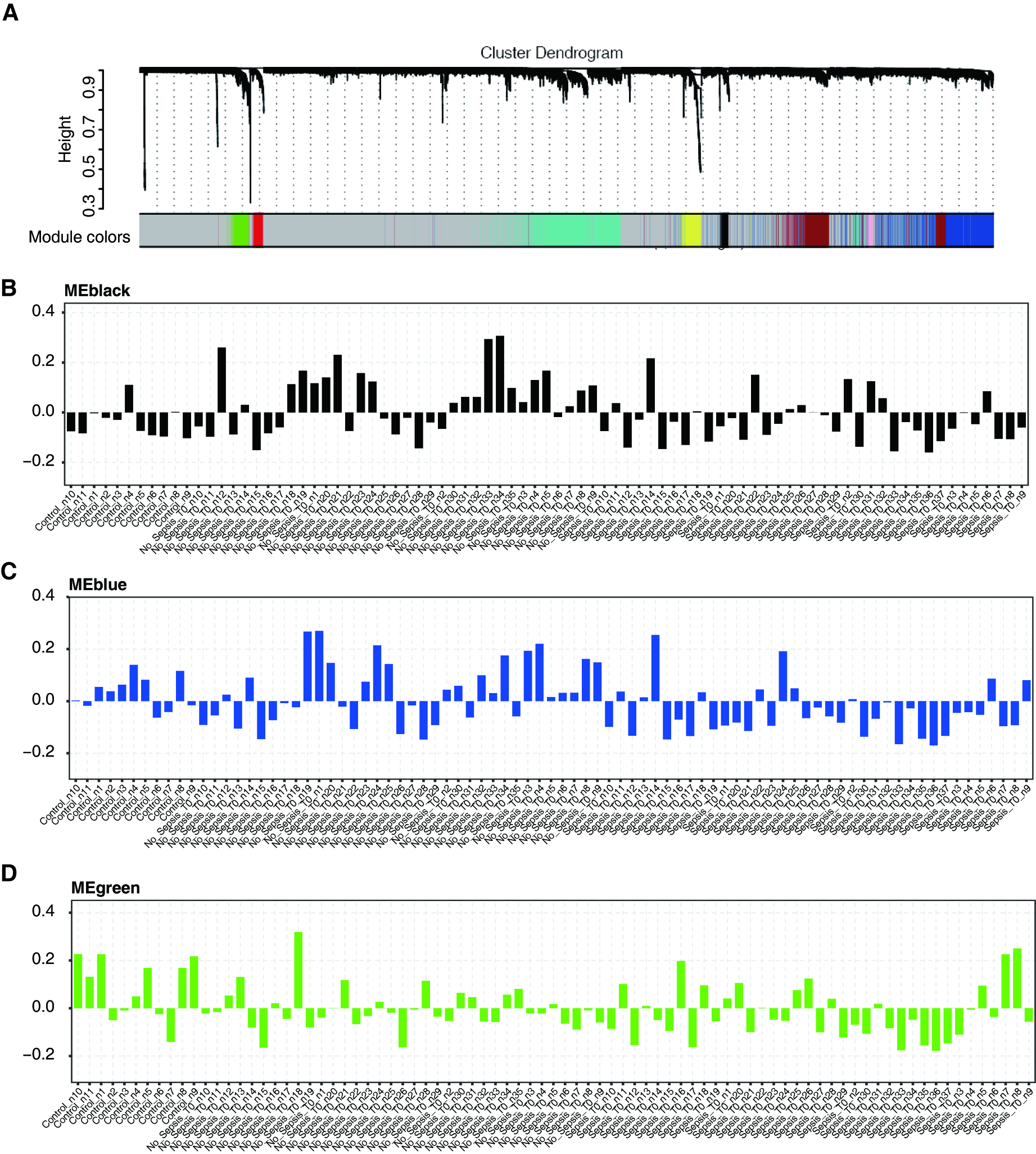

Supplement: Supplementary file 2 [file Image3.tif]

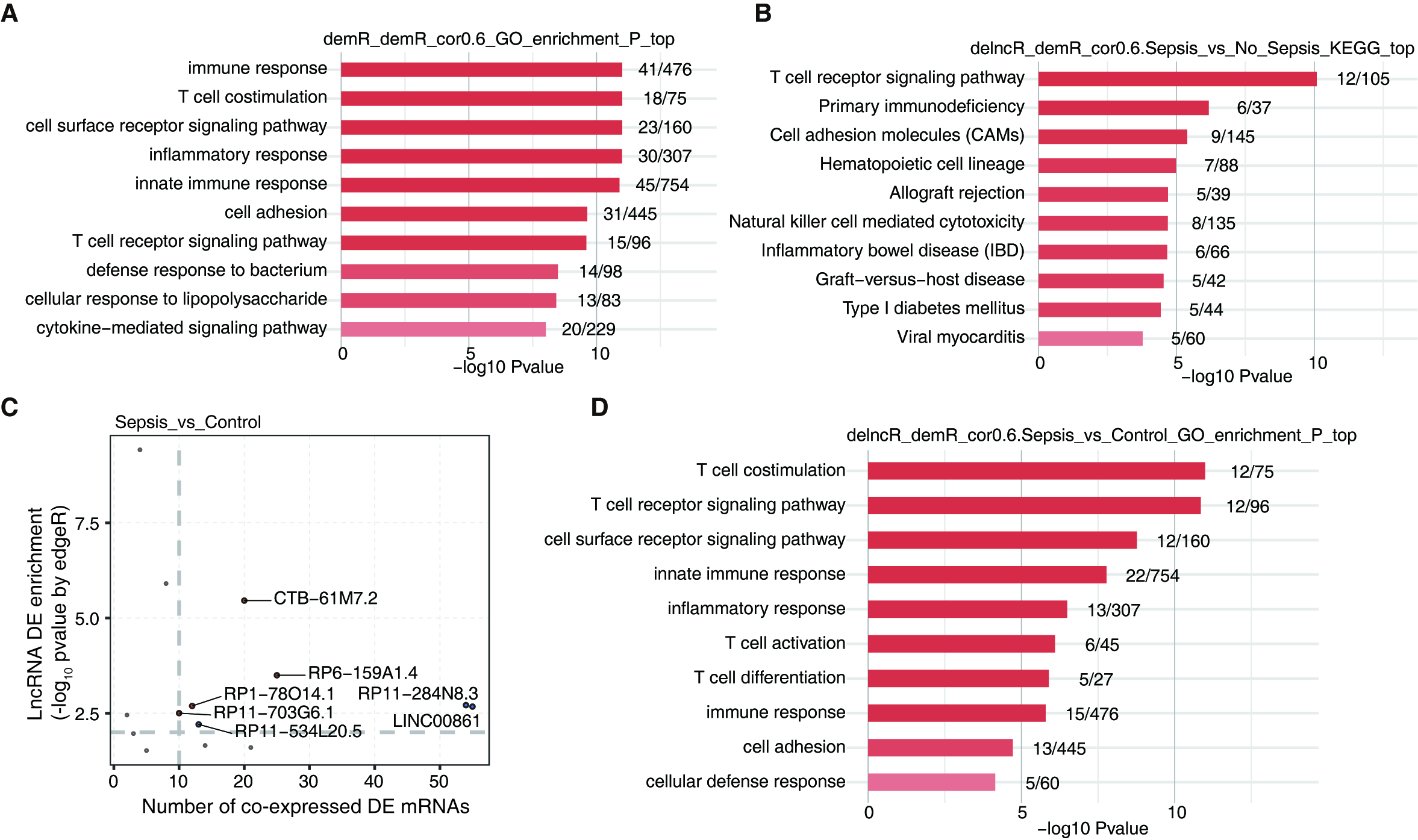

Supplement: Supplementary file 3 [file Image2.TIF]

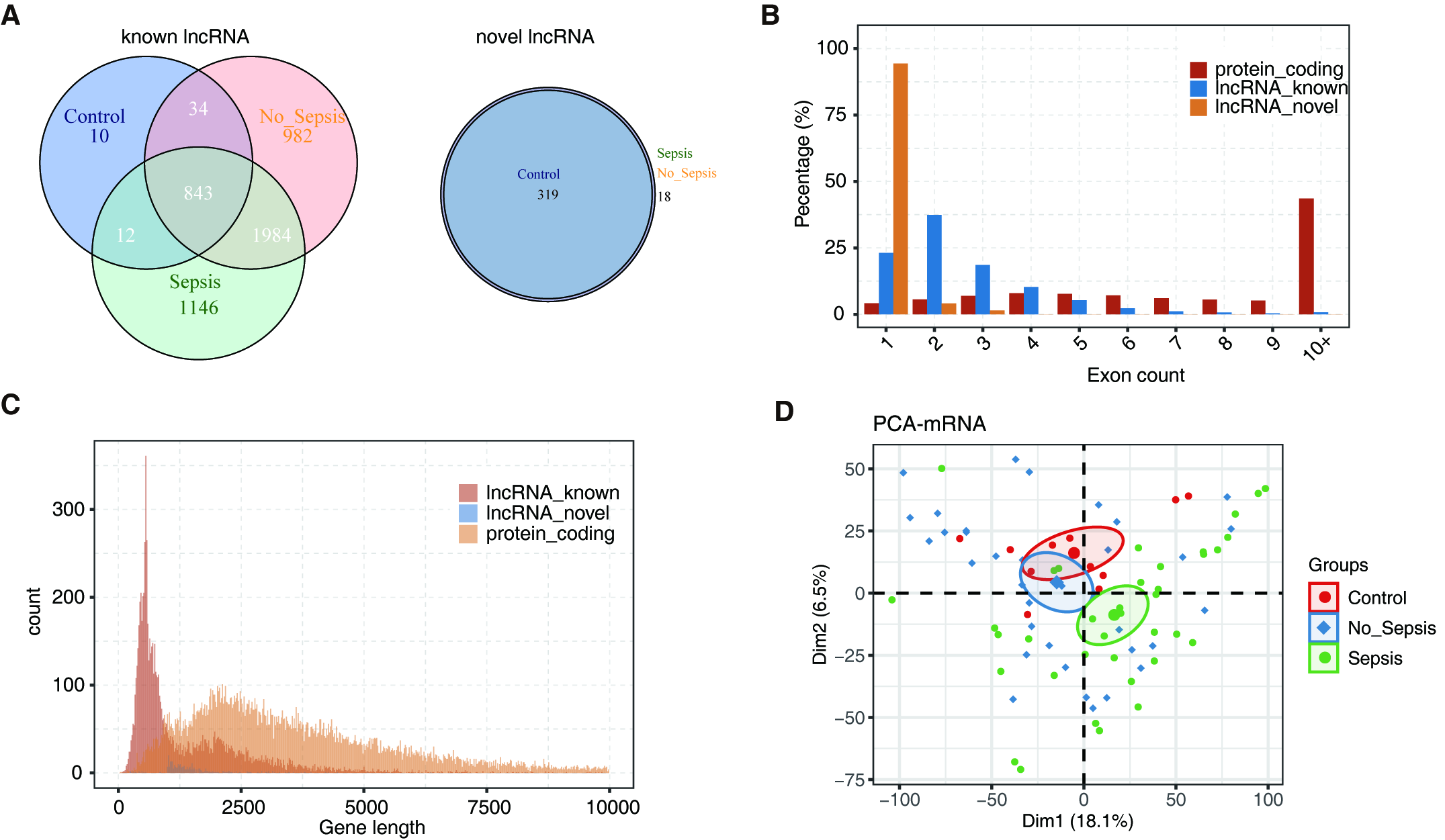

Supplement: Supplementary file 4 [file Image1.TIF]
